# Research on Dynamic Temperature at Outlet of Centrally Staged Combustor Based on TDLAS Technology

**DOI:** 10.3390/s25072256

**Published:** 2025-04-03

**Authors:** Hui Kuang, Xianpu Zhong, Junhao Wei, Fei Xing, Zhenyin Hai

**Affiliations:** Department of Propulsion Engineering, Xiamen University, Xiamen 361005, China; kuanghui@stu.xmu.edu.cn (H.K.);

**Keywords:** centrally staged combustor, TDLAS, high acquisition frequency, high-frequency oscillations

## Abstract

High-frequency oscillations occur in the centrally staged combustor during operation. To effectively suppress them, real-time monitoring of the combustor exit temperature is critical. However, traditional contact temperature measurement methods are inadequate for accurately capturing temperature variations in the turbulent flow field. Tunable Diode Laser Absorption Spectroscopy (TDLAS) with a high acquisition frequency is employed to measure the temperature of the centrally staged combustor, utilizing a non-contact sensing method. The influence of various combustion parameters on the uniformity of combustion within the chamber and the capability of TDLAS to capture temperature data of the combustion chamber under different acquisition frequencies are studied. The results indicate that the staging ratio causes irregular oscillations in the combustion chamber outlet temperature. At an acquisition frequency of 1 kHz, an increase in the staging ratio raises the average temperature at the outlet and slows down the temperature oscillation when other parameters remain constant. At an acquisition frequency of 10 kHz, more small, high-frequency variations in the centrally staged combustor outlet temperature are observed. When the TDLAS system operates at 10 kHz, it can capture more details of the combustion chamber outlet temperature oscillation under the same working conditions and exhibits stronger noise immunity. However, compared with the acquisition frequency of 1 kHz, it cannot sustain long-term measurement.

## 1. Introduction

Modern aero-engines are evolving towards higher thrust-to-weight ratios, lower fuel consumption rates, and higher reliability. As the core component providing power for aero-engines, combustors continue to adopt new structural designs to meet increasingly stringent technical requirements. In addition to fulfilling various combustion performance specifications for the entire engine, low emissions have become a crucial design indicator for advanced aero-engine combustion chambers. This has driven the emergence of centrally staged combustion [1,2]. The combustion scheme of centrally staged combustion combines diffusion combustion and lean premixed combustion. To enhance combustion stability, aerodynamic zoning is employed to generate diffusion flames in localized areas [3]. By adopting the staged combustion concept and optimizing the equivalence ratio in different combustion zones, each section achieves optimal combustion performance. The lean premixed approach enables homogeneous mixing of air–fuel mixtures within the combustion, effectively reducing nitrogen oxides (NOx) emissions [4].

However, in practical applications, the heat release rate pulsation and sound pressure pulsation [5] can lead to uneven combustion [6] and may even give rise to combustion with high-frequency oscillations [7]. These oscillations not only exacerbate pollutant generation but also impose periodic thermal loads and mechanical vibrations on the combustion chamber walls, accelerating the wear and fatigue of the mechanical components and potentially causing irreversible damage to the hardware [8,9]. To suppress these high-frequency oscillations and enable real-time dynamic monitoring of engine health [10], dynamic temperature research on the central combustion chamber has become a critical area of study, playing a significant role in optimizing the combustion process of aerospace burners [11].

To address this challenge, numerous researchers have pioneered studies. Z.Y. Qin et al. [12] from Beihang University predicted high-frequency oscillations by integrating pre-training and transfer learning modes. X.H. Wang [13] from Nanjing University of Aeronautics and Astronautics analyzed pressure pulsation signals using empirical mode decomposition (intrinsic mode function, IMF), providing a data processing method for examining high-frequency oscillations. T. Chao et al. [14] investigated the principles of combustion instability in the combustion chamber using chemiluminescence (CL) and particle image velocimetry (PIV). While existing measurement techniques provide partial temperature insights into centrally staged combustors, the details of parameter changes in the centrally staged combustor are challenging to observe due to their low acquisition frequency [15].

Tunable Diode Laser Absorption Spectroscopy (TDLAS) is an absorption spectroscopy technique that utilizes the wavelength scanning and current modulation characteristics of diode lasers to perform non-contact diagnostics of flow fields [16]. It has become one of the essential measurement methods in aero-engine research. In its early stages, the development and application of TDLAS technology were significantly constrained by limitations in laser devices. However, with continuous advancements in laser technology, the emergence of compact and highly stable Distributed Feedback Lasers (DFB) has driven the rapid progress and widespread adoption of TDLAS [17]. As an effective combustion diagnostic method for aero-engines [18], TDLAS offers a non-intrusive nature compared to contact-based measurement techniques, effectively minimizing disturbances to the flow field’s boundary layer. Its capability for turbulence flame parameter monitoring is enhanced by fast response and high-frequency measurement characteristics [19], enabling precise resolution of complex combustion dynamics [20]. TDLAS technology is utilized to study temperature variations in the centrally staged combustor, explore the fluctuations of uneven combustion phenomena under different combustion conditions, and discuss the data capture capabilities of TDLAS technology at various frequencies.

To further investigate the mechanisms and characteristics of high-frequency oscillations and to obtain more detailed variations in the relevant parameters within the combustion, an in-depth study of the dynamic temperature characteristics at the outlet of the high-frequency centrally staged combustion is conducted. The aim is to provide a data foundation for the health management and technological evolution of engines.

## 2. Materials and Methods

### 2.1. Experimental System

**Figure 1:**
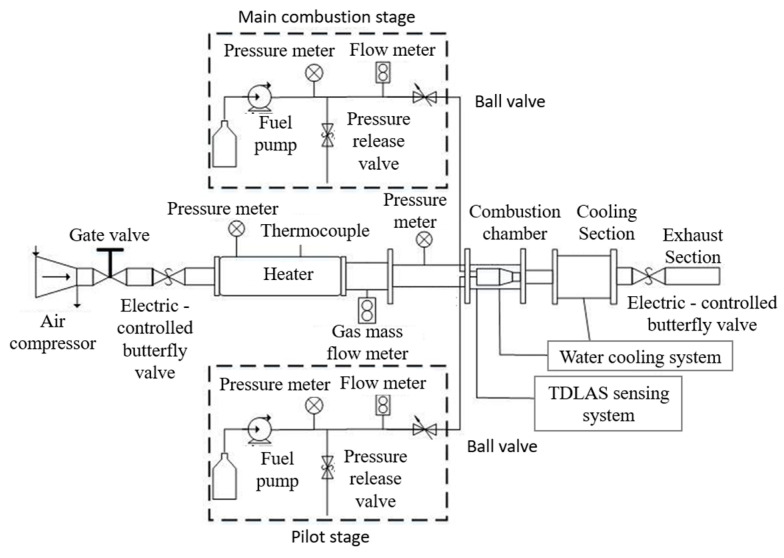
Schematic diagram of the experimental system.

The combustion scheme of the centrally staged combustor included both diffusion combustion and lean premixed combustion [21,22]. Based on the experimental objectives and specific measurement requirements, the construction of the swirling flame experimental system was completed. Figure 1 illustrates the schematic diagram of the overall experimental system.

**Figure 2:**
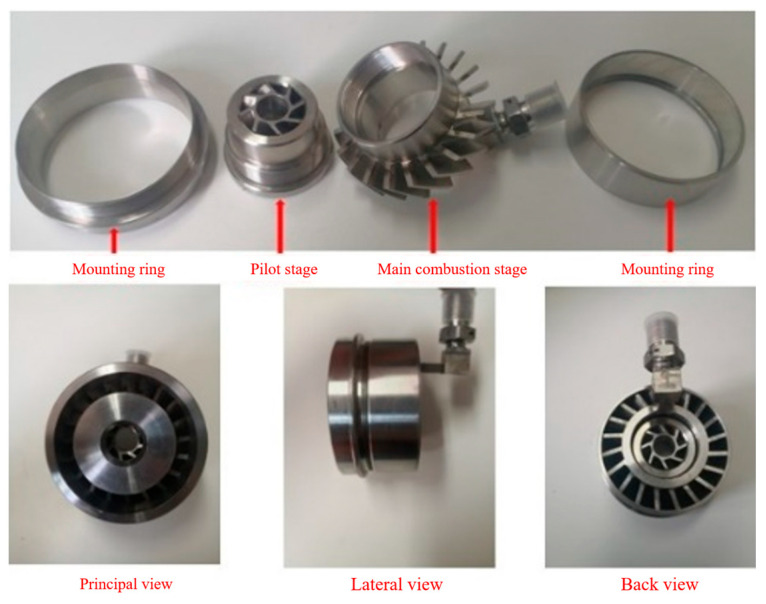
Mounting ring and swirler.

The swirler of the centrally staged combustor comprises three components: the front mounting ring, the rear mounting ring, and the pilot stage, along with the main combustion stage. Figure 2 illustrates the front view, the side view, and the rear view of the swirler. The swirl directions of both the main combustion stage and the pilot stage are identical. All swirlers utilized in this system are axial swirlers. Additionally, the front mounting ring and the rear mounting ring are secured together using right-handed threads, which serve to fix the pilot stage and the main combustion stage in place, ensuring proper alignment and installation within the combustion chamber.

### 2.2. Measurement Specimen

**Figure 3:**
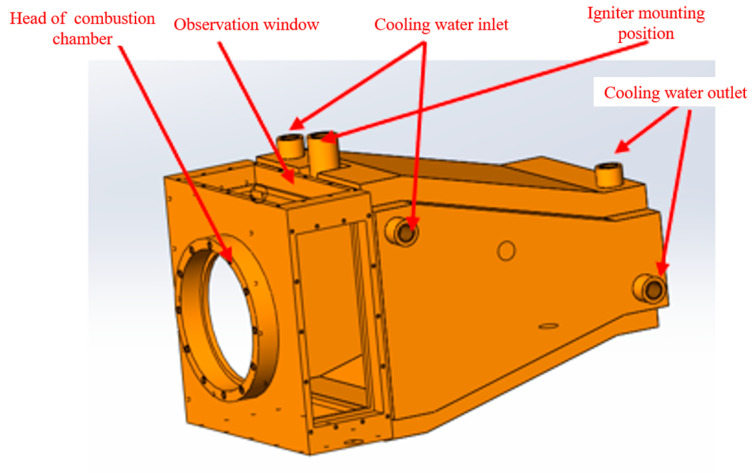
Schematic diagram of the combustion chamber design.

The internal structure of the combustion chamber is illustrated in Figure 3. When utilizing the TDLAS system to measure the temperature of the swirling flame, the primary components to be positioned are the collimator and the photoelectric detector. Considering the subsequent high-frequency oscillations within the combustion chamber, the laser utilized by the TDLAS system is positioned at the outlet of the combustion chamber to investigate the dynamic temperature characteristics at this location. In this experiment, the pilot stage ignition method was employed, with the igniter’s central position located 46 mm from the outlet section of the swirler.

**Figure 4:**
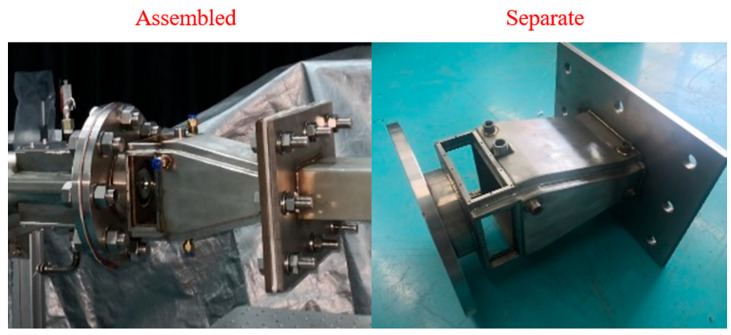
Combustor and installation schematic.

Due to the constraints imposed by the installation position of the igniter and the requirements for the configuration of the observation windows, the dimensions of the observation windows are designed to be 38 mm × 97 mm and 38 mm × 99 mm, respectively. These observation windows enable the measurement of cross-sections ranging from 4 mm to 42 mm from the outlet of the head. High-temperature glass was utilized for the observation windows. For the construction, the 310S stainless steel was selected. This austenitic chromium–nickel stainless steel exhibits excellent oxidation and corrosion resistance, allowing it to operate continuously at elevated temperatures. The wall thickness of the combustion chamber is 3 mm. However, the central temperature within the swirling combustion chamber is relatively high and exceeds the material’s tolerance limit. Therefore, it is essential to cool the wall surface of the combustion chamber. Given the limitations of laboratory conditions, the water cooling method was adopted. Figure 4 illustrates the physical appearance and installation schematic of the combustion chamber.

### 2.3. Sensing System

TDLAS is an optical measurement technique grounded in the Beer–Lambert law [23]. Specifically, when a laser of a particular frequency traverses the target flow field, and this frequency coincides with the transition frequency of a specific molecule within the flow field, the corresponding medium will absorb the laser. Furthermore, the intensity of the laser entering the flow field and the intensity of the light exiting the flow field are related by Equation (1):

(1) $$\ln\left[\frac{I_{t}\left(v\right)}{I_{0}\left(v\right)}\right]=-P\int_{0}^{L}X_{abs}\left(l\right)\cdot S\left(T\left(l\right)\right)\cdot \phi \left(v\right)\cdot dl$$

In the equation, v represents the laser frequency, l represents the total length of the flow field to be measured, $I_{t}$ and $I_{0}$ are the transmitted laser intensity and the incident laser intensity, respectively, P [atm] is the total pressure within the flow field, $S(T\left(l\right))$ [cm^−2^atm^−1^] is the line intensity of the absorption spectral line when the laser triggers molecular transitions, $T\left(l)\right.\;$[K] is the temperature at a certain point, $X_{abs}(l)$ is the gas concentration at a certain point, and ϕ (v) [cm] is the normalized line shape function $\int_{-\infty }^{+\infty }\phi (\nu )d\nu =1$.

Simultaneously, two light rays were allowed to pass through the measured flow field, employing the two-line ratio method for temperature measurement. This method utilizes two spectral lines that follow the same path and determines the temperature by calculating the ratio of the integrated absorption areas of the two spectral lines [24]. The formula for temperature is as follows:

(2) $$T=\frac{\frac{hc}{k}(E_{2}^{\prime \prime }-E_{1}^{\prime \prime })}{\ln\frac{A_{1}}{A_{2}}+\ln\frac{S_{2}(T_{0})}{S_{1}(T_{0})}+\frac{hc}{k}\frac{\left(E_{2}^{\prime \prime }-E_{1}^{\prime \prime }\right)}{T_{0}}}$$

Here, E [cm^−1^] is the energy of the lower energy level of the absorption transition at the same time. The remaining constants are as follows: h[J·s] is the Planck’s constant, c [cm/s] is the speed of light, k [J/K] is the Boltzmann constant, and $T_{0}$ [K] is the reference temperature, which is usually 296 K. $A_{1}$ and $A_{2}$ are the variations in the absorption peak areas of the two spectral lines.

**Figure 5:**
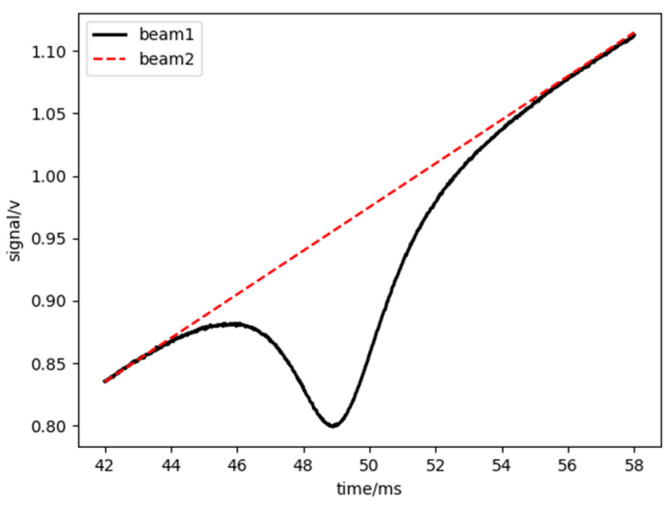
Absorption peak linear fitting graph [26].

It can be observed from this equation that the only unknown quantities are the variations. To analyze this, a linear fit was performed on the absorption peak waveform by selecting several points on either side of the absorption peak to encompass the entire peak [25], as illustrated in Figure 5. Subsequently, these points were substituted into Equation (2) to determine the temperature.

The TDLAS system described employs the absorption spectral lines of water molecules at wavelengths of 1388.1 nm and 1368.6 nm, which are produced by a Distributed Feedback (DFB) laser. These spectral lines are modulated by a sawtooth waveform generated by a signal generator and operate in a time-division multiplexing [27] mode, which enhances signal acquisition. After passing through the laser temperature and current control module, the display positions of the absorption peaks are adjusted to facilitate linear fitting during the subsequent extraction of these peaks. Once the laser is emitted from the collimator, it traverses the measurement area, carrying relevant information about the flow field. The photoelectric detector captures the information transmitted by the laser. Following the conversion of the photoelectric information, the electrical signals are collected and transmitted to a computer, where the next step of parameter calculation is performed.

### 2.4. Experimental Condition

In this experiment, the distance between the outlet of the transition section and the outlet of the combustion chamber is 450 mm. Due to the widths of the collimator and the photoelectric detector, the minimum distance between two adjacent laser beams is 25 mm. Given that the outlet of the transition section is relatively narrow, the single optical path measurement method is employed.

This experiment initially conducted steady temperature measurements using the TDLAS sensing system. Based on the experimental requirements and previous ignition measurement results, a total of 16 operating condition parameters were selected for the experiment. The fuel–air ratio was set at 0.04, while the staging ratios were 100%, 50%, 20%, and 0%, respectively. Additionally, the airflow rates were established at 60.0 g/s, 82.0 g/s, 119.0 g/s, and 182.0 g/s.

The swirler features a two-layer structure comprising the main combustion stage and the pilot stage. These two stages operate with distinct oil circuits to investigate the factors influencing the stratified flame. The staging ratio, as defined in the table, represents the ratio of the fuel flow rate from the pilot stage to the total fuel flow rate. Specifically, a staging ratio of 100% indicates that only the pilot stage supplies fuel, while staging ratios of 50%, 20%, and 10% signify that both the main combustion stage and the pilot stage supply fuel simultaneously. Conversely, the staging ratio of 0% means that only the main combustion stage provides fuel. To explore the characteristics of dynamic temperature under varying fuel–air ratios, the air compressor is adjusted to modify the airflow rate. Additionally, by altering the oil circuit, the characteristics of dynamic temperature under different fuel–air ratio conditions are examined while maintaining a constant airflow rate. Furthermore, by adjusting the oil circuits of both the pilot stage and the main combustion stage, the characteristics of dynamic temperature under various staging ratios are investigated. When adjusting the staging ratio, it is essential to first reduce the fuel supply to the pilot stage after successful ignition, bringing it close to the flameout fuel–air ratio to stabilize the flame. Subsequently, the fuel supply to the main combustion stage can be gradually increased. Finally, further adjustments to the fuel flow rates of both the main combustion stage and the pilot stage are made to satisfy the requirements of the desired operating condition staging ratio.

Next is the 1 kHz single-line measurement of the dynamic temperature at the outlet of the combustion chamber. These data, when combined with the analysis of the experimental phenomena observed during the steady-state measurements, lead us to select the operating condition parameters listed in Table 1 below for the experiment.

The experimental conditions for the 10 kHz measurement experiment are presented in Table 2. Specifically, Working Condition No. 1 corresponds to Serial Numbers 13 of the 1 kHz measurement experiment. Working Conditions No. 2, No. 3, and No. 5 correspond to Serial Numbers 4-3, 4-4, and 4-6 of the 1 kHz measurement experiment, respectively.

## 3. Results

### 3.1. Steady-State Temperature Measurement Results

**Figure 6:**
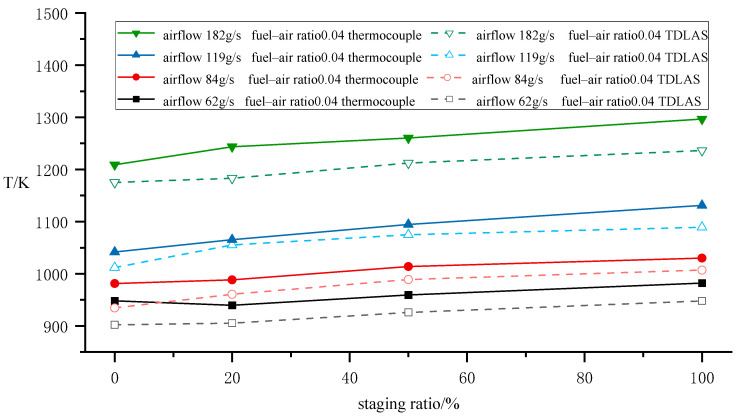
Steady-state average temperature measurement.

The steady-state temperature of the combustor chamber is defined as the average temperature measured. Figure 6 shows the comparison of temperature measurements between the thermocouple and TDLAS under steady-state conditions. The results indicate that at a fixed fuel–air ratio, the outlet temperature increases overall with rising airflow. When the airflow increases while maintaining the same fuel–air ratio, the corresponding fuel flow rate also increases. The enhanced fuel injection into the combustion extends the fuel spray field downstream and enlarges the reaction zone, thereby elevating the outlet temperature with increased airflow. Under constant airflow conditions, the outlet temperature exhibits a slight upward trend as the staging ratio increases, though the magnitude of this increase is limited. Calculations of thermocouple temperature values across different flow rates reveal a maximum temperature deviation of 4.98% between the 100% staging ratio and 0% staging ratio configurations.

The temperature measurement results obtained by TDLAS exhibited a consistent trend compared with thermocouple measurements. But the TDLAS results were generally lower than the thermocouple temperature values, with relative errors all below 4.78%. This discrepancy arises because safety considerations for the collimating lens and photodetector required their placement to maintain a certain distance from the flow field, resulting in partial inclusion of non-combustion zones along the actual measurement path. As TDLAS measures path-averaged temperature, the presence of these non-combustion zones leads to an overall underestimation.

### 3.2. Measurement Results Under the Acquisition Frequency of 1 kHz

**Figure 7:**
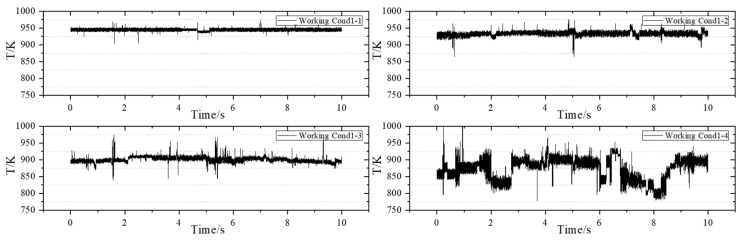
Measurement results of the airflow condition group of 62.0 g/s.

**Figure 8:**
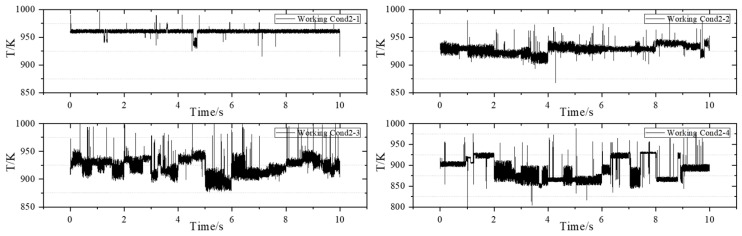
Measurement results of the airflow condition group of 84.0 g/s.

**Figure 9:**
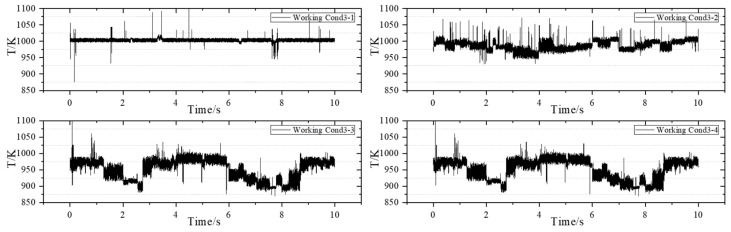
Measurement results of the airflow condition group of 119.0 g/s.

The next operating condition is applied, and the measurement frequency of the TDLAS measurement system adjusted to 1 kHz. The measurement results are as follows: Figure 7 corresponds to the operating condition with an airflow rate of 62.0 g/s, Figure 8 corresponds to the operating condition with an airflow rate of 84.0 g/s, and Figure 9 corresponds to the operating condition with an airflow rate of 119.0 g/s.

Working Conditions 1-1, 2-1, and 3-1 are analyzed. Under a constant staging ratio, as the airflow rate increases, the average outlet temperature of the swirling flame is recorded at 944.8 K, 960.5 K, and 1003.2 K, respectively. In comparison to other scenarios where only the airflow rate is increased while maintaining a constant staging ratio, the average temperature at the outlet of the combustion chamber exhibits an upward trend. This phenomenon occurs because the increase in airflow rate enhances fuel atomization, resulting in more uniform combustion and a corresponding rise in the average temperature at the outlet of the combustion chamber.

The operating condition group with an airflow rate of 62 g/s is analyzed separately. Under the condition of a constant airflow rate, as the staging ratio decreases, the average temperatures recorded are 944.8 K, 933.0 K, 900.4 K, and 874.4 K, respectively. Additionally, when comparing other operating conditions, a decrease in the staging ratio at a constant airflow rate results in a slight downward trend in the outlet temperature. The temperature amplitudes at the outlet of the combustion chamber are 70.1 K, 112.0 K, 132.1 K, and 190.3 K, respectively. It can be seen that when the staging ratio is 100%, the swirling flame combustion is stable, the temperature oscillation is small, and the temperature field is relatively stable. When the staging ratio is 50%, the swirling flame combustion is generally stable, but compared with the operating condition with a staging ratio of 100%, the temperature oscillation is larger, and the overall measured temperature value is also smaller than that of the operating condition with a staging ratio of 100%. As the airflow rate increases, the outlet temperature gradually increases, and under the operating condition with a large airflow rate, the oscillation of the outlet temperature is more obvious than that with a small airflow rate, and the fluctuation range of the outlet temperature value is relatively large.

**Figure 10:**
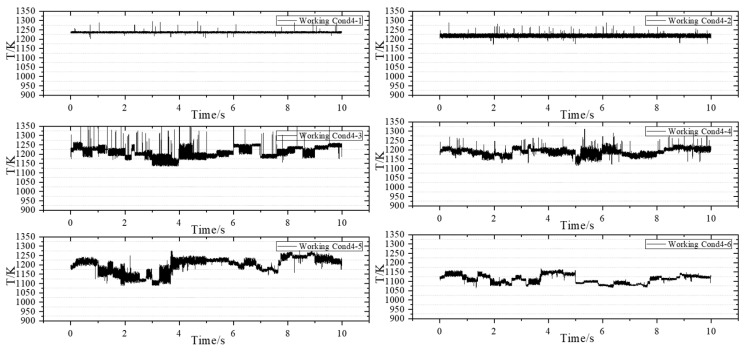
Measurement results of the airflow condition group of 182.0 g/s.

Subsequently, the experiment is conducted under Working Condition 4 to obtain dynamic temperature measurements at 1 kHz. The purpose of implementing Working Condition 4 is to align with the measurement conditions at a frequency of 10 kHz, allowing for a comparison and observation of the detailed changes in temperature at the outlet of the combustion chamber. Additionally, this study aims to investigate the impact of reducing fuel–air ratio on temperature uniformity at the outlet of the combustion chamber while keeping other parameters constant. Figure 10 illustrates the operating condition with an airflow rate of 182.0 g/s.

When other parameters remain constant and the fuel–air ratio is simultaneously reduced, the average temperature at the outlet of the combustion chamber decreases. Further analysis is conducted on the phenomenon of temperature oscillation within the combustion chamber. During the experimental process, it was observed that when the pilot stage operates independently, the flame burns stably, and no significant high-frequency oscillation was observed.

When comparing the four sets of data with a fuel-to-air ratio of 0.04, it is observed that as the airflow rate increases, the temperature oscillation range under high-staging-ratio operating conditions expands further. Over time, the outlet temperature exhibits drastic changes, which aligns with the phenomena observed in the experiment. A similar oscillation is noted under high-staging-ratio operating conditions. Additionally, it is evident that the changes and oscillations in temperature are neither regular nor periodic. Furthermore, the sudden spikes or drops in temperature values within a single cycle become increasingly pronounced. When the staging ratio falls below 20%, the overall measurement results display violent fluctuations that are irregular, consistent with the oscillation phenomenon observed in the experiment.

### 3.3. Measurement Results Under the Acquisition Frequency of 10 kHz

**Figure 11:**
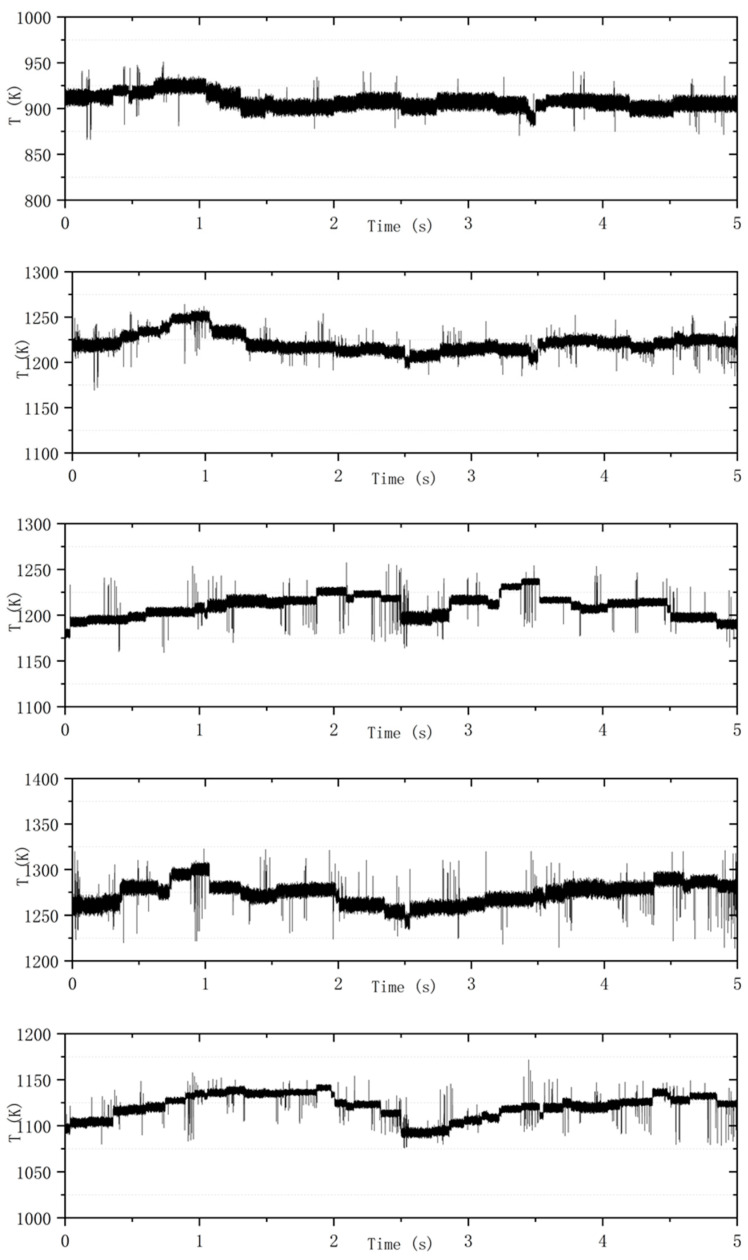
Dynamic 10 kHz temperature measurement results.

The acquisition frequency of the TDLAS measurement system is 10 kHz. The results of the measurements are presented below. As illustrated in Figure 11, the results correspond to Working Conditions 1 through 5, respectively.

By analyzing the data collected by the Tunable Diode Laser Absorption Spectroscopy (TDLAS) system at a frequency of 10 kHz, the variation in the average temperature at the outlet of the combustion chamber is further validated. When comparing the results of the TDLAS system at acquisition frequencies of 1 kHz and 10 kHz across four corresponding operating conditions, the average temperatures recorded at 1 kHz are 900.4 K, 1211.5 K, 1189.3 K, and 1112.5 K, respectively. In contrast, the average temperatures recorded at 10 kHz are 907.9 K, 1221.3 K, 1209.8 K, and 1121.1 K, respectively. The similarity in the average temperatures not only demonstrates the reliability of the testing system but also facilitates the comparison of relevant data.

When the TDLAS system operates at an acquisition frequency of 1 kHz, it demonstrates a certain dynamic collection capability, allowing it to capture the high-frequency temperature oscillations at the outlet of the combustion chamber and to reflect some detailed temperature changes. In contrast, when the system collects data at 10 kHz, it can capture more intricate variations at the outlet of the combustion chamber, providing improved continuity and enhanced noise resistance. However, due to the data storage limitations of the data register, an acquisition frequency of 10 kHz is not suitable for long-term measurements, whereas the 1 kHz frequency can sustain longer operational periods. Consequently, the data collected at 10 kHz are utilized to supplement the data gathered at 1 kHz.

Under the same Working Condition 1, when the data acquisition frequency is set to 10 kHz, a distinct periodic temperature change is observed around 0.6 s, following a high-frequency, small-amplitude decrease. This phenomenon is also noted in Working Conditions 2, 3, and 4 at the same frequency of 10 kHz. Conversely, at an acquisition frequency of 1 kHz, this change is only observed at approximately 2.6 s in Working Condition 4. This suggests that the data capture ability of the 10 kHz frequency is stronger when facing detailed changes. Meanwhile, during the measurement at 1 kHz, the amplitudes measured under the four working conditions are 45.7 K, 97.3 K, 75.0 K, and 81.9 K, respectively; during the measurement at 10 kHz, the amplitudes measured under the four working conditions are 38.3 K, 51.2 K, 58.1 K, and 49.1 K, respectively. Under the same combustion working conditions, when requiring temperature data with a longer duration, an acquisition of 1 kHz is used; when requiring short-term precise temperature data, an acquisition frequency of 10 kHz is used.

In general, under various working conditions, the presence of the staging ratio leads to irregular oscillations in the temperature of the flow field at the outlet of the combustion chamber. During the experiment, the pilot stage was initially employed for ignition. Subsequently, while maintaining a constant total fuel amount, the fuel supply to the pilot stage was reduced, and the fuel supply to the main combustion stage was increased to adjust the fuel–air ratio to the experimental working conditions. The phenomena observed during the experiment align with the measurement results, indicating that the staging ratio induces specific high-frequency oscillations in the flame. This oscillation simultaneously drives vibrations throughout the entire combustion chamber and the connecting sections, resulting in increasingly intense noise during the experiment, which resembles oscillatory behavior.

## 4. Conclusions

In order to study the effectiveness of the TDLAS technology in dealing with the special application scenarios of the combustion chamber, a centrally staged combustor was set up, and the TDLAS system was utilized for dynamic temperature measurement. The following conclusions were drawn:

(1) The TDLAS system, at an acquisition frequency of 1 kHz, exhibits a certain dynamic collection capacity, capable of reflecting some detailed temperature variations. In contrast, the data collected at 10 kHz can reveal more intricate temperature changes, with enhanced continuity and noise resistance. The combined utilization of 10 kHz and 1 kHz frequencies enables a more comprehensive reflection of the dynamic temperature characteristics of the temperature field.
(2) The outlet temperature change of the centrally staged combustor is affected by multiple combustion parameters. Under the condition that the fuel–air ratio and the staging ratio remain the same, increasing the airflow rate will enhance the degree of fuel atomization, make the combustion more uniform, and increase the outlet temperature, while the temperature oscillation phenomenon is significantly enhanced. Under the condition that the airflow and the fuel–air ratio remain unchanged, in order to alleviate the high-frequency temperature oscillations, the staging ratio of the centrally staged combustor can be increased, and at the same time, the average temperature at the outlet of the combustion chamber will be slightly increased. This provides a data basis for adjusting the engine thrust and combustion efficiency.

## Figures and Tables

**Table 1:** The 1 kHz experimental working condition.

S/N	Airflow (g/s)	Fuel Flow (g/s)	Oil-Gas Ratio	Staging Ratio (%)
1-1	62.0	2.48	0.04	100
1-2				50
1-3				20
1-4				0
2-1	84.0	3.36	0.04	100
2-2				50
2-3				20
2-4				0
3-1	119.0	4.76	0.04	100
3-2				50
3-3				20
3-4				0
4-1	182.0	7.28	0.04	100
4-2				50
4-3				20
4-4				10
4-5				0
4-6		6.37	0.035	20

**Table 2:** The 10 kHz experimental working condition.

S/N	Airflow (g/s)	Fuel Flow (g/s)	Incoming Pressure (MPa)	Oil–Gas Ratio	Staging Ratio (%)
1	61.0	2.44	0.1	0.04	20
2	178.0	7.12	0.1	0.04	20
3	178.0	7.12	0.1	0.04	10
4	187.0	7.48	0.21	0.04	20
5	178.0	6.23	0.1	0.035	20

## Data Availability

The data that support the findings of this study are available on request from the corresponding author. The data are not publicly available due to privacy or ethical restrictions.
